# Magnesium Oxychloride Cement Composites with MWCNT for the Construction Applications

**DOI:** 10.3390/ma14030484

**Published:** 2021-01-20

**Authors:** Michal Lojka, Anna-Marie Lauermannová, David Sedmidubský, Milena Pavlíková, Martina Záleská, Zbyšek Pavlík, Adam Pivák, Ondřej Jankovský

**Affiliations:** 1 Department of Inorganic Chemistry, Faculty of Chemical Technology, University of Chemistry and Technology, Technická 5, 166 28 Prague 6, Czech Republic; Michal.Lojka@vscht.cz (M.L.); Anna-Marie.Lauermannova@vscht.cz (A.-M.L.); David.Sedmidubsky@vscht.cz (D.S.); 2 Department of Materials Engineering and Chemistry, Faculty of Civil Engineering, Czech Technical University in Prague, Thákurova 7, 166 29 Prague 6, Czech Republic; milena.pavlikova@fsv.cvut.cz (M.P.); martina.zaleska@fsv.cvut.cz (M.Z.); pavlikz@fsv.cvut.cz (Z.P.); adam.pivak@fsv.cvut.cz (A.P.)

**Keywords:** composites, magnesium oxychloride cement, multi-walled carbon nanotube (MWCNT)

## Abstract

In this contribution, composite materials based on magnesium oxychloride cement (MOC) with multi-walled carbon nanotubes (MWCNTs) used as an additive were prepared and characterized. The prepared composites contained 0.5 and 1 wt.% of MWCNTs, and these samples were compared with the pure MOC Phase 5 reference. The composites were characterized using a broad spectrum of analytical methods to determine the phase and chemical composition, morphology, and thermal behavior. In addition, the basic structural parameters, pore size distribution, mechanical strength, stiffness, and hygrothermal performance of the composites, aged 14 days, were also the subject of investigation. The MWCNT-doped composites showed high compactness, increased mechanical resistance, stiffness, and water resistance, which is crucial for their application in the construction industry and their future use in the design and development of alternative building products.

## 1. Introduction

The interest in alternative construction materials has been growing continuously in recent decades. Environmentally sustainable composites are being studied more and more and used in practice to reduce the man-made emissions from the industry processes and thus the gradual deterioration of the environment. Large amounts of greenhouse gases (GHG), mainly carbon dioxide, are released during the production of Portland cement (PC) and PC-based composite materials [1]. The search for an alternative for PC has been previously described in the literature, showing the great potential of reactive magnesia-based cement [2,3,4]. Lately, the interest in reactive magnesia-based cement is mainly focused on magnesium oxychloride cement (MOC).

Magnesium oxychloride cement, also known as Sorel cement, is a term describing cementitious material formed in the MgO-MgCl_2_-H_2_O system [5]. Depending on the reaction temperature and the molar ratio of magnesium oxide and magnesium chloride used as raw materials, four phases are formed in the synthesis. Phase 3 (3Mg(OH)_2_·MgCl_2_·8H_2_O) and Phase 5 (5Mg(OH)_2_·MgCl_2_·8H_2_O) are formed at ambient temperature [6,7,8], whereas Phase 2 (2Mg(OH)_2_·MgCl_2_·4H_2_O) and Phase 9 (9Mg(OH)_2_·MgCl_2_·4H_2_O) are formed at temperatures above ~100 °C [9,10,11]. Previous studies have shown the unique properties of MOC, such as its fire resistance and its resistance to abrasion, as well as its outstanding mechanical properties [12,13,14]. In comparison to PC, the setting time of MOC is fractional, making it usable as a material that is suitable for quick repairs [15]. The main disadvantage of MOC is its poor water resistance. After interacting with water, MOC structure is destroyed and the high mechanical strength is lost [16]. This problem can be resolved by using various additives, which improve the water resistance. The use of soluble phosphates [17], organic acids [18], sewage sludge ash [19], or fly ash [20] for this purpose has been previously described in the literature [21,22,23].

The main aspect making MOC environmentally sustainable is its so-called CO_2_-neutrality. When calcining magnesite, which is the main procedure in the production of raw materials for MOC, the temperature is much lower than the calcination temperature of calcite being used in the production process of PC [24,25,26]. In addition, as previously described in the literature [27], MOC can absorb the atmospheric CO_2_. MOC can be used as a matrix in composite materials with many different fillers, such as silica sand, fly ash, porcelain waste, and others [28,29]. Moreover, additives improving the specific properties of MOC can be used, where those improving the water resistance are mentioned above. Another approach is based on using nanoadditives, namely carbon-based nanoadditives, which improve the mechanical properties of MOC, as has been recently pointed out in the literature [30].

Carbon-based nanomaterials have outstanding mechanical, chemical, and physical properties, which make them applicable as additives in construction materials. Graphene and its derivatives represent the group of 2D carbon nanomaterials that have been previously studied as additives used to improve the mechanical properties of the matrix [31,32,33,34]. Single- and multi-walled carbon nanotubes (SWCNTs and MWCNTs) are representatives of 1D carbon nanomaterials. They are tubular carbon macromolecules containing carbon in the sp^2^ hybridization. SWCNTs consist of a single sheet of graphite being formed into a seamless tubular shape, while MWCNTs consist of many of these nanotubes constituting concentric circular shapes, similar to the annual rings in a tree trunk [35,36]. CNTs exhibit outstanding mechanical, chemical, thermal, and electrical properties [37]. This nanoadditive has been previously used in composite materials to improve their mechanical and electrical properties [38,39]. One of the most useful traits of CNTs is their ability to improve the compressive strength of the composite material. Another quality, which can be improved by the use of CNTs, is the water resistance. This attribute can be quite important when designing novel construction materials [40,41,42,43,44]. The problem of MOC’s poor water resistance was mentioned above, and a solution of it could be possibly hidden in the use of small amounts of CNTs.

In this study, a composite material based on MOC with MWCNTs was synthesized and characterized. Two different amounts of CNTs were applied to optimize the most suitable composition of the binder. The synthesized composites were compared to the conventional MOC to evaluate the beneficial effect of the CNTs. The prepared specimens were analyzed in terms of their phase and chemical composition, morphology, thermal behavior, and mechanical properties to help describe their behavior in various environments and applications. This research indicates a possible solution of the poor water resistance of MOC-based materials in general.

## 2. Experimental Section

### 2.1. Materials and Synthetic Procedures

**Figure 1:**
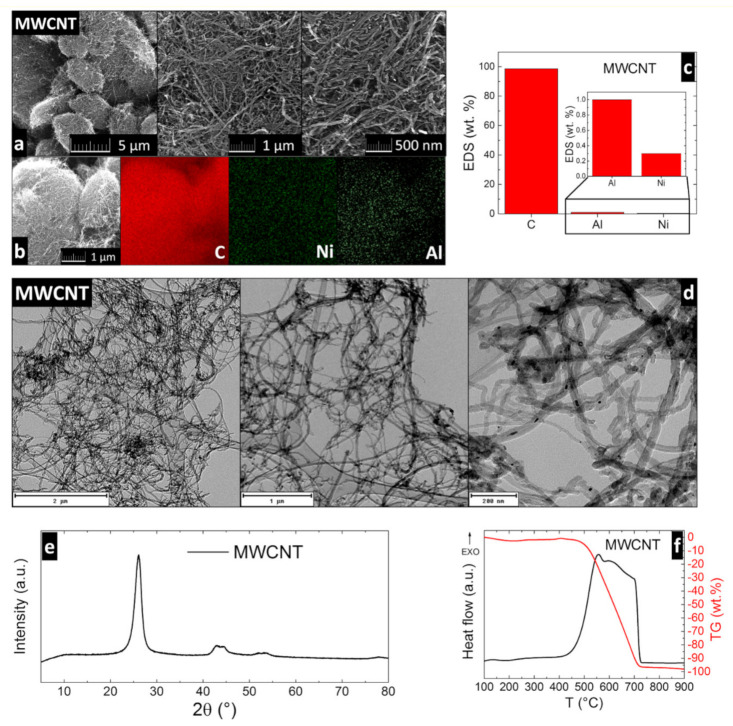
Analysis of multi-walled carbon nanotubes (MWCNTs): (**a**) SEM micrographs; (**b**) elemental maps of MWCNTs obtained by EDS; (**c**) quantity of the elements from EDS; (**d**) TEM micrographs; (**e**) diffraction pattern of MWCNTs; and (**f**) thermal behavior obtained by simultaneous thermal analysis (STA).

The following chemicals were used for the synthesis: MgCl_2_∙6H_2_O (>99%, Penta s.r.o., Prague, Czech Republic) and MgO (>80%, Styromagnesit Steirische Magnesitindustrie Ltd., Oberdorf, Austria). The caustic magnesia powder contained 80.4 wt.% of MgO, 4.3 wt.% of SiO_2_, 5.0 wt.% of CaO, 5.8 wt.% of Al_2_O_3_, 3.9 wt.% of Fe_2_O_3_, and less than 1 wt.% of sulfates. Its BET surface area was 26.07 m^2^∙g^−1^, and the particle size distribution parameters were *d*_50_ = 41.71 μm and *d*_90_ = 65.87 μm. MWCNTs (TNIM8) were purchased from TimesNano (Chengdu, China) with declared purity > 95%. These MWCNTs were analyzed in detail before their use in composites. The morphology was analyzed using SEM. The micrographs (see Figure 1a) show the typical tubular structure of MWCNTs. The chemical composition was determined by the EDS method. The elemental maps obtained by EDS, as well as the graph showing the quantities of the present elements, are shown in Figure 1b,c. Both the maps and the quantitative analysis showed high purity of MWCNTs, with more than 98.7 wt.% content of carbon and only less than 1 wt.% of aluminum and ~0.3 wt.% of nickel. Detailed morphology was studied by TEM. The micrographs (see Figure 1d) show the tubes with width in the order of tens of nanometers and lengths up to 20 μm. The phase composition was determined using X-ray diffraction, showing the specific reflection of MWCNTs at 2θ = 26.1° (see Figure 1e). The thermal behavior of MWCNTs was studied using simultaneous thermal analysis (STA) (Figure 1f). The sample was heated to 900 °C in dynamic air atmosphere. During this process, an exothermic effect occurred at temperatures between 410 °C and 740 °C, which is connected to the oxidation of the carbon nanotubes. This effect was also accompanied with a significant weight loss, which is clearly visible from the TG curve.

The mix proportions of the chemicals used for the synthesis of composites are given in Table 1. The magnesium chloride solution was prepared by dissolving MgCl_2_∙6H_2_O in the tap water. MWCNTs were then sonicated in the part of the prepared MgCl_2_ solution for 15 min, and the obtained suspensions were used for mixing with MgO powder. The samples were casted into molds with dimensions of 40 mm × 40 mm × 160 mm, and the samples were demolded after 24 h. The composites were then cured for the next 13 days at laboratory temperatures; *T* = (23 ± 2) °C in air atmosphere, RH = (50 ± 5)%. The resulting samples were termed MOC-CNT-R (reference sample), MOC-CNT-0.5 (0.5 wt.% of MWCNT), and MOC-CNT-1 (1 wt.% of MWCNT). Let us note that the amount of MWCNT was calculated in relation to the sum of solid raw materials (MgO and MgCl_2_∙6 H_2_O).

### 2.2. Analytical Techniques

To determine the phase composition, X-Ray powder diffraction (XRD) was carried out. Bruker D2 Phaser (Bruker, Karlsruhe, Germany), the powder diffractometer with Bragg Brentano geometry, applying CuKα radiation (λ = 0.15418 nm, U = 30 kV, I = 10 mA) and a rotation (5 r/min), was used. The used angular range was set to 5–80°, and the step size was set to 0.02025° (2θ). The measured data evaluation, as well as the semi-quantitative analysis, was performed using the X’Pert Highscore Plus software (v. 3.0.5).

The study of the surface morphology was performed using scanning electron microscopy (SEM) with the Tescan MAIA 3 apparatus (Brno, Czech Republic). The elemental composition and elemental maps were obtained by means of an energy dispersive spectroscopy (EDS) analyzer (X-Max150) with a 20 mm^2^ SDD detector (Oxford instruments) and AZtecEnergy software 3.0. To manipulate the sample, and in order to ensure the conductivity, carbon conductive tape was used. The setting for both experiments (SEM and EDS) was the same—the electron beam was set to 10 kV. The EDS was performed from the fracture surface. All samples were sputtered by 10 nm of gold in order to increase the surface conductivity (to avoid charging).

The thermal behavior of the samples was analyzed using simultaneous thermal analysis (STA) with the Setsys Evolution apparatus from Setaram (Geneva, Switzerland) with the temperature ramp up to 900 °C at a heating rate of 10 K·min^−1^. The measurements were performed in a dynamic helium atmosphere with a flow rate of 50 mL·min^−1^. In order to analyze the gases being evolved during the heating process, the mass spectrometer OmniStarTM from Pffeifer Vacuum (Aßlar, Germany) was used.

The 14-day laboratory cured composites were examined. Experiments, including assessment of the structural, mechanical, thermal, and hygric properties, were performed. In these tests, at least five samples of each composite were tested. The presented values represent the mean values taken from the data obtained for the particular samples.

Among the structural parameters of the hardened composites, bulk density, specific density, and total open porosity were investigated. The bulk density was obtained from the measurement of the dry sample mass and its volume, as prescribed in the EN 1015-10 [45]. The specific density was measured using a helium automatic pycnometer Pycnomatic ATC (Thermoscientific, Milan, Italy), equipped with the automatic temperature control. Based on the knowledge of the bulk density and specific density, the total open porosity of researched materials was determined [46,47]. The combined expanded uncertainty of the fundamental structural parameter assessment was 1.4%, 1.2%, and 2.0% for the bulk density, specific density, and total open porosity tests, respectively. Helium pycnometers Pascal 140 and Pascal 440 (Thermo Fisher Scientific, Waltham, MA, USA) were applied in the pore size distribution analysis. The fragments of originally casted samples, with a typical mass of ~2 g, were measured. To avoid inhomogeneity of samples in contact with the iron mold surface, the inner part of the original prisms was fragmented.

Flexural strength, compressive strength, and the dynamic Young’s modulus were the tested mechanical parameters. The standard EN 1015 -11 [48] was followed in the strength tests. The 40 mm × 40 mm × 160 mm prisms were used in flexural strength testing. The halves of broken prisms were then subjected to the compression load. The loaded cross-sections were 40 mm × 40 mm. The expanded combined uncertainty of the realized strength tests was 1.4%. The dynamic Young’s modulus was tested using a non-destructive ultrasonic pulse velocity test on Vikasonic apparatus (Schleibinger Geräte, Buchbach, Germany). The Young’s modulus was determined with the expanded combined uncertainty of 2.3%.

Since MOC-based materials have been reported to be vulnerable to water-induced damage [49,50], the effect of the CNT incorporation into the MOC matrix on water imbibition and transport was investigated. The tested hygric parameters were 24-h water absorption and water absorption coefficient. In the water absorption measurement, the EN 13755 [51] was followed. The uncertainty of this test was 1.2%. Based on the free water intake experiment, organized according to the standard EN 1015-18 [52], the water absorption coefficient was calculated using a one-tangent method. The uncertainty in the water absorption coefficient assessment was 1.2%.

Identification of thermo-physical parameters of the examined composites was performed on a transient place source technique using Hot Disk TPS 1500 (Hot Disk AB, Göteborg, Sweden). Before the measurement itself, the probed samples were dried in a vacuum drier at 60 °C. The hot disk testing was conducted at controlled laboratory temperature *T* = (23 ± 2) °C. As declared by the Hot Disk producer, the accuracy of the measurement was better than 5% and the reproducibility was better than 1%.

## 3. Results and Discussion

**Figure 2:**
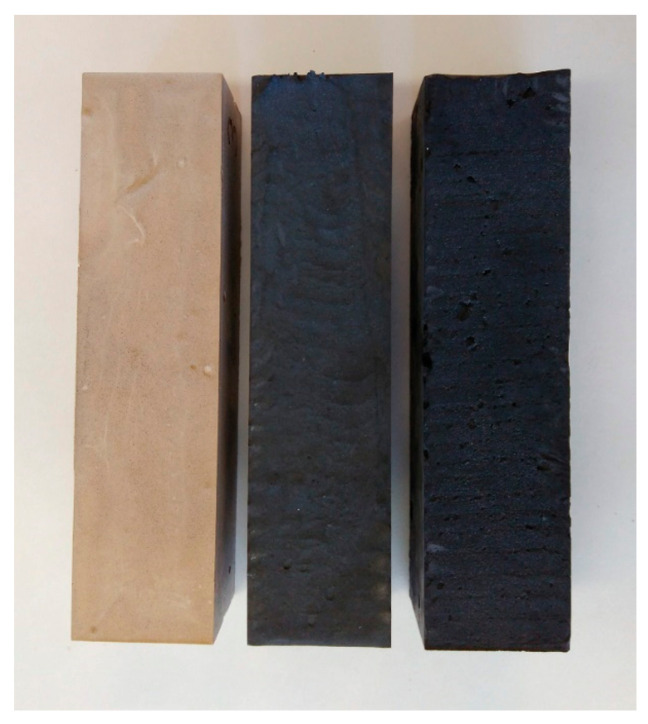
Photograph of the prepared composites MOC-CNT-R (**left**), MOC-CNT-0.5 (**middle**), and MOC-CNT-1 (**right**).

Composites composed of MOC and MWCNT were prepared and characterized in detail. The samples MOC-CNT-R, MOC-CNT-0.5, and MOC-CNT-1 are shown in Figure 2.

**Figure 3:**
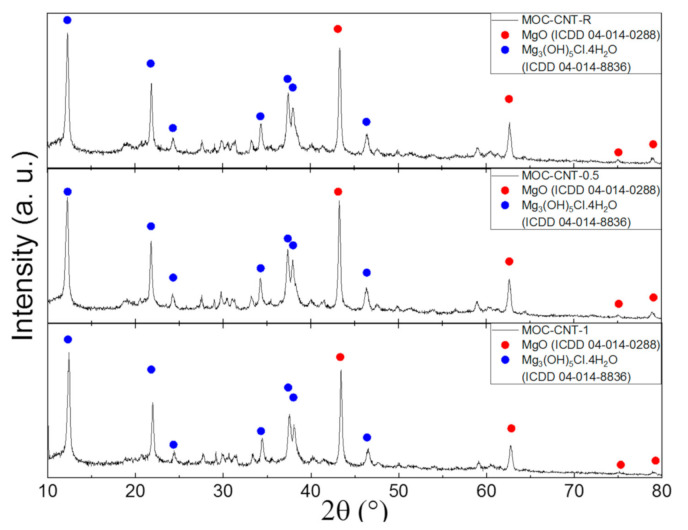
Diffraction patterns of the samples MOC-CNT-R, MOC-CNT-0.5, and MOC-CNT-1.

First, the phase composition of all the samples was studied by XRD. The diffraction patterns of all samples show the presence of the MOC phase 5 (ICDD 04-014-8836) and MgO (ICDD 04-014-0288). The presence of unreacted magnesium oxide is not problematic, since the residual oxide acts as a filler in this composite material. The MWCNTs were not visible in the diffraction pattern due to their small amount in the sample, in comparison to the other phases. The diffraction patterns of all samples can be seen in Figure 3. These results are in good agreement with the theoretical calculation based on mass balance, where 68% of MgO should react and the remaining 32% should remain unreacted in the form of a filler. The semi-quantitative analysis showed 31 wt.% content of MgO for MOC-CNT-R, 23 wt. % content of MgO for MOC-CNT-0.5, and 28 wt.% content of MgO for MOC-CNT-1.

**Figure 4:**
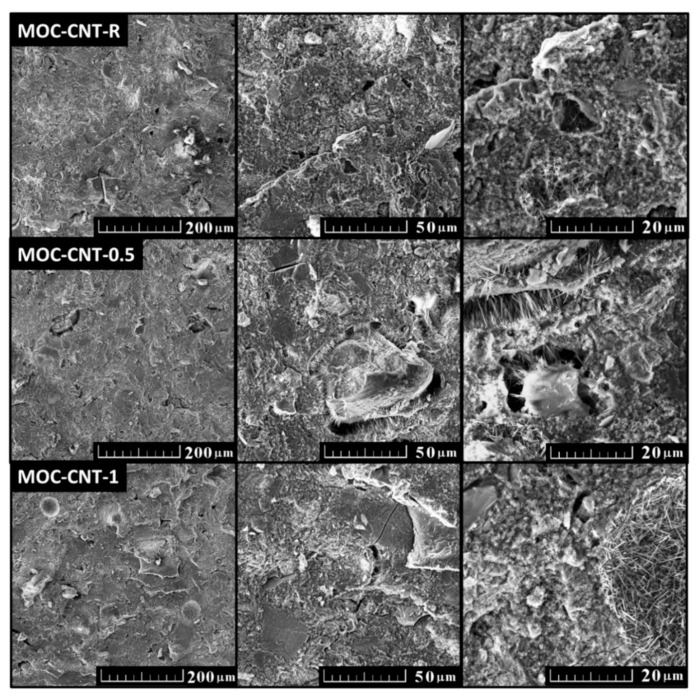
SEM micrographs of the samples MOC-CNT- R, MOC-CNT-0.5, and MOC-CNT-1.

The microstructure of the samples was analyzed using SEM. All samples showed the presence of needle-shaped crystals, which are typical for MOC. The typical dimensions of the crystals were 1–3 µm in length and ~0.5 µm in thickness. The SEM micrographs of the composites are displayed in Figure 4.

The chemical composition of the samples was determined using EDS, confirming the expected composition. Apart from magnesium, oxygen, chlorine, and carbon, traces of iron, silicon, and calcium were also detected. These impurities originated from MgO powder. While magnesium, oxygen, and chlorine were homogeneously distributed on the respective maps, carbon maps revealed carbon-rich areas, suggesting insufficient homogenization of the mixture before casting or, alternatively, a high tendency of MWCNTs to agglomeration. Some carbon was also detected in the reference sample. This might be due to the formation of chlorartinite on the sample surface, which has been previously described in the literature [27]. The elemental maps are shown in the supporting information (Figure S1).

**Figure 5:**
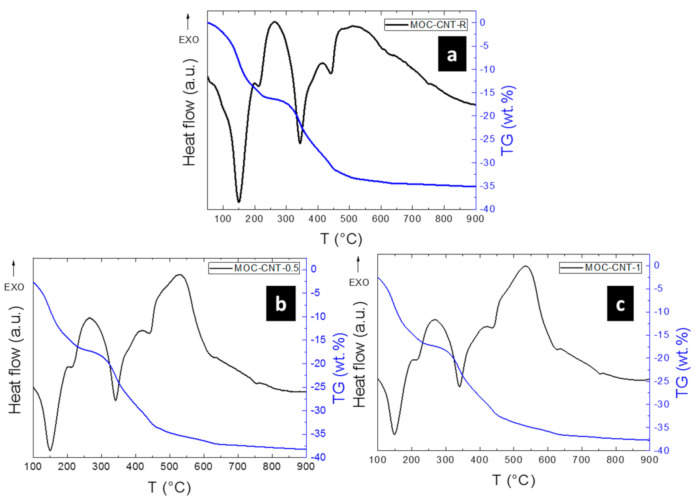
STA of (**a**) MOC-CNT-R, (**b**) MOC-CNT-0.5, and (**c**) MOC-CNT-1.

The thermal behavior of the samples was analyzed using STA (Figure 5). The samples were heated from ambient temperature to 900 °C, and their decomposition was observed. Two main effects were identified during the heating—the oxidation of MWCNTs (between 450 °C and 600 °C) and the gradual decomposition of MOC phase 5 throughout the heating process, whose individual steps have been already described in the literature [22]. This process mainly consists of a release of crystalline water at lower temperatures and hydrochloric acid at higher temperatures. These endothermal effects are clearly visible on the DTA curve, along with the corresponding weight decrease seen on the TG curve. After the heating, the resulting solid phase after the decomposition is pure magnesium oxide.

The effect of the CNT admixture is apparent from Table 2, where the structural and mechanical parameters of the investigated composites are summarized. The bulk density, specific density, and total open porosity were reduced by the incorporation of CNT into the composite mixture. The most notable drop in porosity was obtained for the MOC-CNT-0.5 composite, whose porosity was decreased by approximately 19% compared to the reference material MOC-R. The total open porosity of MOC-R-1 was slightly higher than that of MOC-CNT-0.5, which was affected by the formation of CNT agglomerates during the material mixing and setting [53]. On the other hand, the porosity of MOC-CNT-1 was still about 5% lower in comparison with the control material. The effect of CNT doping on the basic structural parameters is visualized in the supporting information (Figure S2).

All examined materials exhibited high mechanical resistance, which was in agreement with the previously reported results of the mechanical testing of MOC-based products. [20,54,55] The excellent mechanical properties of CNT [56,57] are manifested by the improved mechanical resistance and stiffness of CNT-doped composites. In comparison to the reference material, the flexural strength increased by about 11% for MOC-CNT-0.5 and by ~17% for the MOC-CNT-1 composite. The increase in the compressive strength was approximately 6% and 12% for MOC-CNT-0.5 and MOC-CNT-1, respectively. The stiffness of CNT-enriched materials was also higher than that of the MOC-R reference material. The improvement in mechanical resistance depended, among other effects, on the material porosity. However, in the evaluated mechanical resistance of MOC-CNT materials, the high strength of CNT prevailed over the porosity effect.

**Figure 6:**
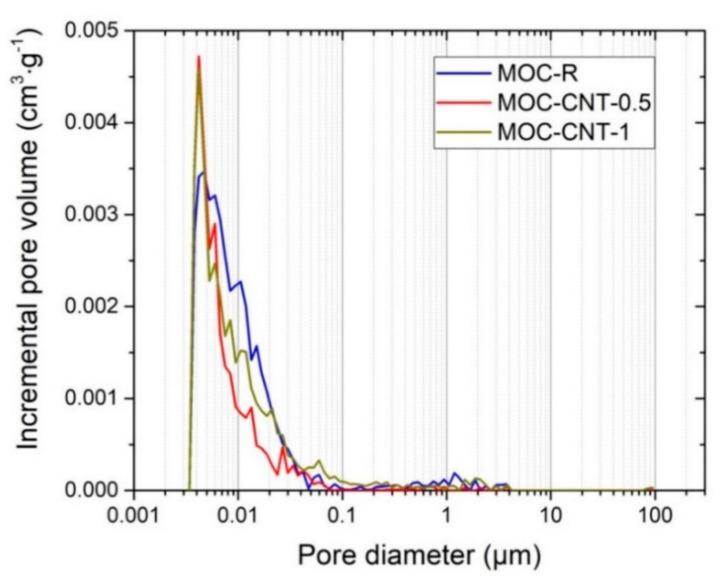
Incremental pore volume distribution of the investigated composites.

**Figure 7:**
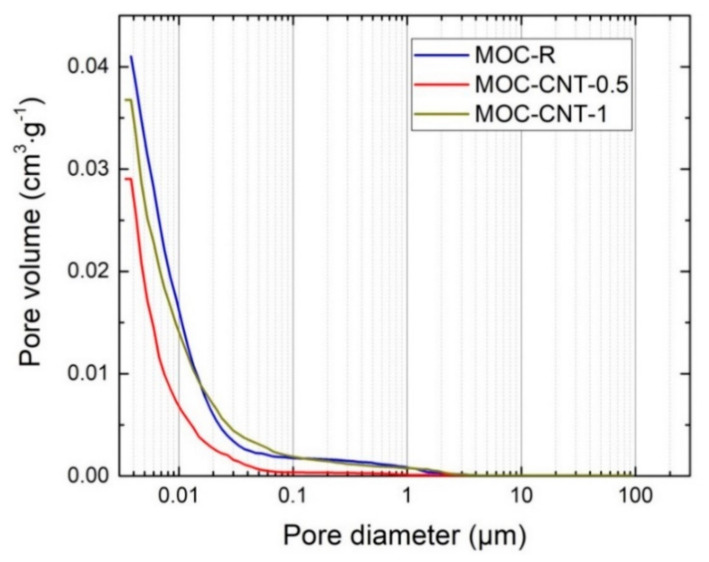
Cumulative pore volume of the investigated composites.

Not only the total open porosity, but also the average pore diameter, was decreased by the use of CNT, which documented the refinement of porous space of the CNT-doped composites. The average pore diameters were the following: 0.0076 μm (MOC-R), 0.0060 μm (MOC-CNT-0.5), and 0.0071 μm (MOC-CNT-1). Accordingly, the respective median pore diameters were 0.0078 (MOC-R), 0.0056 MOC-CNT-0.5), and 0.0070 μm (MOC-CNT-1). The incremental and cumulative curves of pore size distribution are graphed in Figure 6 and Figure 7.

The hygrothermal function of the developed composites is apparent in Table 3. The hydrophobic performance of CNT [58] in the contact with water molecules significantly decreased, as documented on both observed hygric parameters. This result is very promising because of high susceptibility of MOC materials to moisture damage. As the water ingress was strongly reduced by the use of CNT in composite mixtures, the better durability of the final materials can be expected. The difference in the thermal conductivity of the studied materials was small, but the effect of highly conducting CNT was very visible. Typically, the higher dosage of CNT increased the thermal conductivity of MOC-CNT-0.5 and MOC-CNT-1 materials as a result of the enormous thermal conductivity of CNT [59]. Accordingly, the volumetric heat capacity of MOC-CNT-1 composites was the highest.

## 4. Conclusions

In this contribution, the impact of multi-walled carbon nanotubes on a magnesium oxychloride-based binder was studied. Samples of MWCNT-doped MOC composite were prepared in various weight ratios and characterized in terms of their phase and chemical composition, morphology, thermal behavior, and mechanical properties. We decided to use MWCNTs due to their higher tensile strength and larger diameter compared to SWCNTs. We believe that MWCNTs are more suitable for interaction/cross-linking with MOC. The data gained from the conducted analyses and tests enabled us to point out the following most substantial findings:

(i). Stable and durable MOC phase 5, formed properly and with no crystalline impurities, were present in the sample (except for the parent MgO acting as a filler);
(ii). The structure of the developed composites was highly compacted without any visible defects;
(iii). The thermal behavior of the hardened materials was presumably comparable to the behavior of MOC phase 5 alone, with the exception of MWCNT oxidation, which was observed in the temperature region between 450–600 °C;
(iv). The MWCNT-doped composites exhibited increased mechanical resistance and stiffness, which was due to the lower porosity, average particles size, and excellent mechanical parameters of MWCNT;
(v). The incorporation of MWCNTs resulted in greatly reduced water ingress. which is positive for material durability in the presence of moisture;
(vi). The heat transport and storage were moderately increased by the incorporation of MWCNTs into the composites.

Based on the obtained results, it can be concluded that the developed composites enriched with MWCNTs possess interesting functional and technical properties, which give them a potential for a wide variety of applications in the construction industry and, in some specific cases, enable us to substitute Portland cement-based products.

The high mechanical resistance of the researched composites represents a good prerequisite for their use in combination with a high volume of inorganic fillers, such as diatomite, foam glass granules, perlite, expanded clay granulate, etc. This will aim to achieve high quality and high-performance materials for specific use.

In consideration of the improved mechanical resistance and greatly dropped water absorption, the economic viability of the use of MWCNTs in the doping of the MOC matrix can be considered as very promising. The incorporation of 0.5 wt.% and 1 wt.% of MWCNTs in composite composition enhanced the compressive strength of approximately 11% and 17%, whereas the price increase of the produced materials was approximately 0.05 USD/dm^3^ and 0.10 USD/dm^3^, respectively. Moreover, for the MWCNT-doped materials, the reduced water softening can be anticipated.

## Figures and Tables

**Table 1:** Mix proportions of composites (g).

Mixture	Mass (g)			
	MgO	MgCl_2_∙6H_2_O	Water	MWCNT
MOC-CNT-R	1318.6	584.1	388.3	0
MOC-CNT-0.5	1318.6	584.1	388.3	9.5
MOC-CNT-1	1318.6	584.1	388.3	19.0

**Table 2:** Basic structural and mechanical properties of the tested composites.

Material	MOC-R	MOC-CNT-0.5	MOC-CNT-1
Bulk density *ρ_b_* (kg.m^−3^)	1913 ± 27	1907 ± 27	1885 ± 26
Specific density *ρ_s_* (kg∙m^−3^)	1975 ± 24	1957 ± 24	1943 ± 23
Total open porosity *Ψ* (%)	3.14 ± 0.06	2.55 ± 0.05	2.99 ± 0.06
Flexural strength *f_f_* (MPa)	14.1 ± 0.2	15.7 ± 0.2	16.5 ± 0.2
Compressive strength *f_c_* (MPa)	71.4 ± 1.0	77.5 ± 1.1	80.2 ± 1.1
Young’s modulus *E_d_* (GPa)	24.7 ± 0.6	25.8 ± 0.6	26.1 ± 0.6

**Table 3:** Hygric and thermal parameters of the tested composites.

Parameter	MOC-R	MOC-CNT-0.5	MOC-CNT-1
24-h water absorption (%)	1.19 ± 0.01	1.06 ± 0.01	1.05 ± 0.01
Water absorption coefficient (kg∙m^−2^∙s^−1/2^)	0.0016	0.0010	0.0008
Thermal conductivity (W∙m^−1^∙K^−1^)	1.519	1.531	1.618
Thermal diffusivity × 10^−5^ (m^2^∙s^−1^)	0.772	0.811	0.802
Volumetric heat capacity × 10^5^ (J∙m^−3^∙K^−1^)	1.967	1.888	2.010

## Data Availability

The data presented in this study are available on request from the corresponding author. Additional data are present in Supporting Information file.

## Supplementary Materials

The following are available online at https://www.mdpi.com/1996-1944/14/3/484/s1, Figure S1: Elemental maps of the samples MOC-CNT-R, MOC-CNT-0.5 and MOC-CNT-1, Figure S2: Reduction of the macrostructural parameters (Total open porosity in red, bulk density in gold and specific density in blue) of MOC-CNT-0.5 and MOC-CNT-1 composites due to the CNT admixture.
