# Supplementary material for: Magnesium Oxychloride Cement Composites with MWCNT for the Construction Applications
Source: Materials (Basel). 2021 Jan 20;14(3):484. doi: 10.3390/ma14030484 (PMC7864339; doi:10.3390/ma14030484)
Supplement: Supplementary file 1 [file materials-14-00484-s001.pdf]

materials

Article

SUPPORTING INFORMATION

Magnesium oxychloride cement composites with MWCNT for the construction applications

Michal Lojka[1], Anna-Marie Lauermannová[1], David Sedmidubský[1], Milena Pavlíková[2], Martina Záleská[2], Zbyšek Pavlík[2], Adam Pivák[2] and Ondřej Jankovský*[1]

[1] Department of Inorganic Chemistry, Faculty of Chemical Technology, University of Chemistry and Technology, Technická 5, 166 28 Prague 6, Czech Republic; Michal.Lojka@vscht.cz (M.L.); Anna-Marie.Lauermannova@vscht.cz (A.-M.L.); David.Sedmidubsky@vscht.cz (D.S.); ondrej.jankovsky@vscht.cz (O.J.)

[2] Department of Materials Engineering and Chemistry, Faculty of Civil Engineering, Czech Technical University in Prague, Thákurova 7, 166 29 Prague 6, Czech Republic; milena.pavlikova@fsv.cvut.cz (M.P.); martina.zaleska@fsv.cvut.cz (M.Z.); adam.pivak@fsv.cvut.cz (A.P.); pavlikz@fsv.cvut.cz (Z.P.)

* Correspondence: ondrej.jankovsky@vscht.cz; Tel.: +420-220-44-2002

Citation: Lojka, M.; Lauermannová, A.-M.; Sedmidubský, D.; Pavlíková, M.; Záleská, M.; Pavlík, Z.; Pivák, A.; Jankovský, O. Magnesium oxychloride cement composites with MWCNT for the construction applications. *Materials* **2021**, *14*, 484. https://doi.org/10.3390/ma14030484

Received: 26 December 2020
Accepted: 18 January 2021
Published: 20 January 2021

Publisher's Note: MDPI stays neutral with regard to jurisdictional claims in published maps and institutional affiliations.

Copyright: © 2021 by the author. Licensee MDPI, Basel, Switzerland. This article is an open access article distributed under the terms and conditions of the Creative Commons Attribution (CC BY) license (http://creativecommons.org/licenses/by/4.0/).

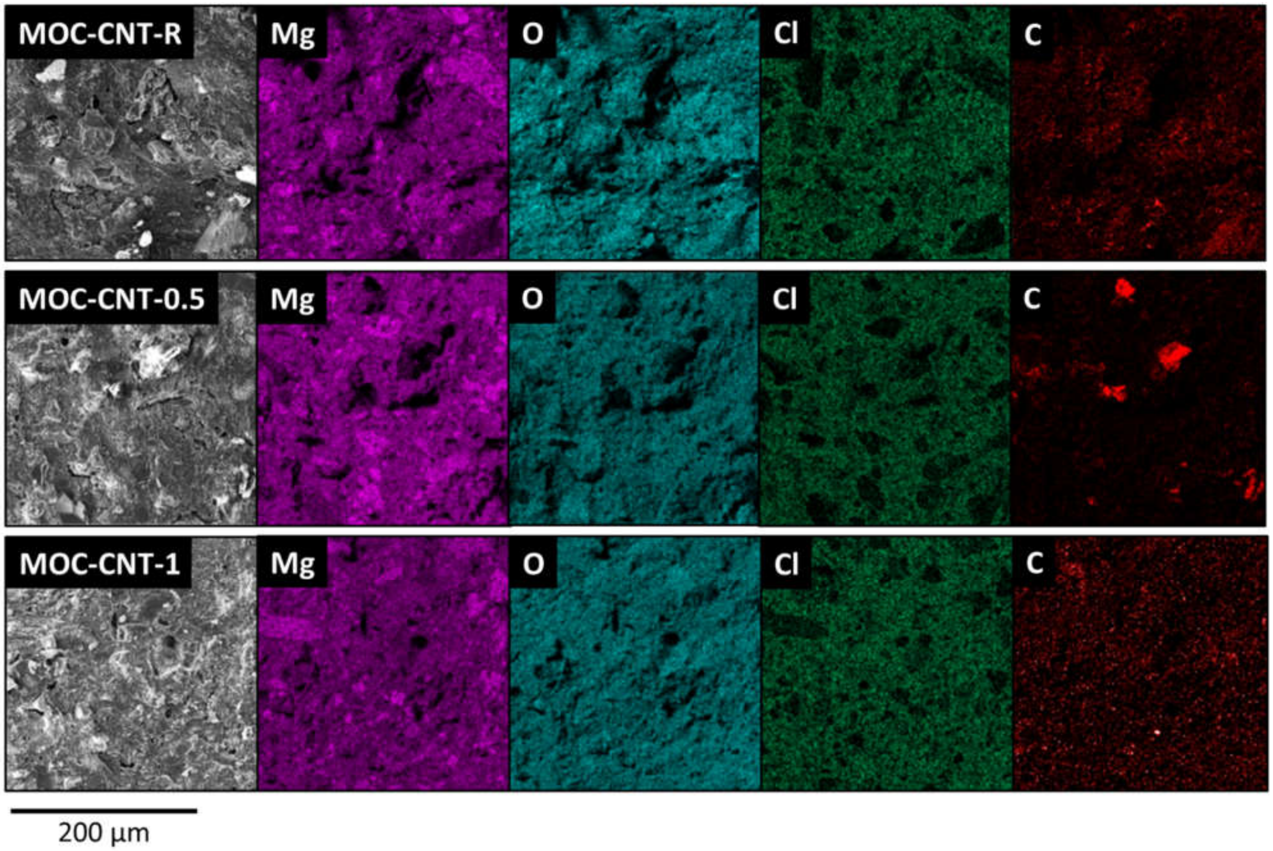

Figure S1. Elemental maps of the samples MOC-CNT-R, MOC-CNT-0.5 and MOC-CNT-1.

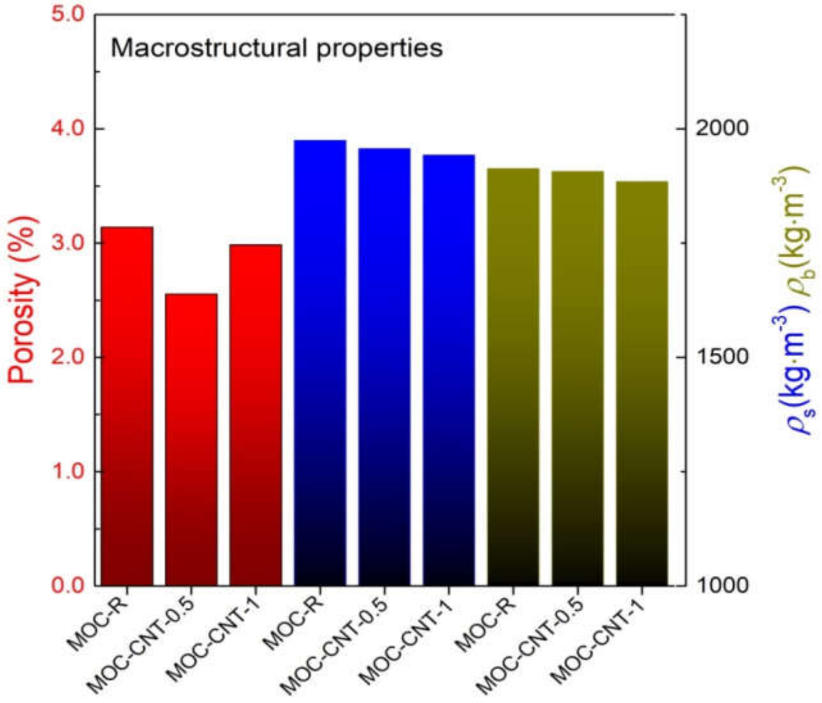

Figure S2. Reduction of the macrostructural parameters (Total open porosity in red, bulk density in gold and specific density in blue) of MOC-CNT-0.5 and MOC-CNT-1 composites due to the CNT admixture.
